# Supplementary material for: Patterns of Intron Gain and Loss in Fungi
Source: PLoS Biol. 2004 Nov 30;2(12):e422. doi: 10.1371/journal.pbio.0020422 (PMC532390; doi:10.1371/journal.pbio.0020422)
Supplement: Table S1 — Also available at http://genes.mit.edu/NielsenEtAl/. (4.3 MB ZIP). [file pbio.0020422.st001.zip › NielsenEtAl/html/425.html]

AN4218.1.NCU02003.1.MG03641.1.FG08811.1


```
 CLUSTAL W (1.82) Multiple Sequence Alignments - Introns Inserted


Sequence 1: NCU02003.1	482 aa
Sequence 2: FG08811.1	460 aa
Sequence 3: AN4218.1	470 aa
Sequence 4: MG03641.1	473 aa
Alignment Length: 503 aa
Number Identitical Residues: 357 aa
Alignment Score (without introns) 14876


MG03641.1 	----------------------MGNKEKSHLN0VVVIG2HVDSGKSTT0T--------~G
NCU02003.1	MSSLSIRSRAQQSLYASFANNCHSKEDKTHIN~VVVIG~HVDSGKSTT~T--------~G
FG08811.1 	----------------------MGKEEKTHLN~VVVIG~HVDSGKSTT0T--------~G
AN4218.1  	--------------------MINSKEDKTHIN~IVVIG~HVDSGKSTT~TGKTSSHPT0G
          	                       .:::*:*:* :**** ********* *..::: .: *

MG03641.1 	HLIYKLKGIDQRTIEKYEK0EAAELGKGSFKYAWVLDKLKAERERGITIDIALWKFETAK
NCU02003.1	HLIYKCGGIDKRTIEKFEK0EAAELGKGSFKYAWVLDKLKAERERGITIDIALWKFETPK
FG08811.1 	HLIYQCGGIDKRTIEKFEK0EAAELGKGSFKYAWVLDKLKAERERGITIDIALWKFETPR
AN4218.1  	HLIYKCGGIDQRTIEKFEK~EAAELGKGSFKYAWVLDKLKSERERGITIDIALWKFQTPK
          	****:  ***:*****:** ********************:***************:*.:

MG03641.1 	YQVTVID1APGHRDFIKNMITGTSQADCAILVIGAGTGEFEAGISKDGQTREHALLAFTL
NCU02003.1	YYVTVID1APGHRDFIKNMITGTSQADCAILIIAAGTGEFEAGISKDGQTREHALLAYTL
FG08811.1 	YYVTVID1APGHRDFIKNMITGTSQADCAILIIAAGTGEFEAGISKDGQTREHALLAYTL
AN4218.1  	YEVTVID1APGHRDFIKNMITGTSQADCAILIIASGTGEFEAGISKDGQTREHALLAFTL
          	* ***** ***********************:*.:**********************:**

MG03641.1 	GVRQLIVAVNKMDTAKWAQSRYDEIVKETSNFLKKIGFNPDSVPFVPISGFNGDHMISES
NCU02003.1	GVKQLIVAINKMDTTQWSQTRFEEIIKETKNFIKKVGYNPAGVAFVPISGFNGDNMLEP-
FG08811.1 	GVKNLIVAINKMDTTKWSEARYQEIIKETSSFIKKVGYNPKAVAFVPISGFNGDNMLTA-
AN4218.1  	GVRQLIVALNKMDTCQWSEARYNEIVKETSGFIKKVGYNPKAVAFVPISGFHGDNMLEP-
          	**::****:***** :*:::*::**:***..*:**:*:** .*.*******:**:*:   

MG03641.1 	ADIKGNISPNAPWYKGWTKTVNKDGKKEKVIGGASLQDAIDDVTPPTRPTDKPLRLPLQD
NCU02003.1	-------STNCPWYKGWEKET-KAGK----ATGKTLLEAIDAIEPPKRPTDKPLRLPLQD
FG08811.1 	-------STNCPWYKGWEREI-KSGK----LSGKTLLEAIDSIEPPKRPNDKPLRLPLQD
AN4218.1  	-------SANCPWYKGWEKET-KAGK----VTGKTLLEAIDAIEPPVRPSNKPLRLPLQD
          	       *.*.****** :   * **      * :* :*** : ** **.:*********

MG03641.1 	VYKIGGIGTVPVGRIETGILKPGMVVTFAPANVTTEVKSVEMHHQQLPEGVPGDNVGFNV
NCU02003.1	VYKIGGIGTVPVGRIETGVLKPGMVVTFAPSNVTTEVKSVEMHHEQLAQGVPGDNVGFNV
FG08811.1 	VYKIGGIGTVPVGRIETGIIKPGMVVTFAPSNVTTEVKSVEMHHEQLTEGQPGDNVGFNV
AN4218.1  	VYKISGIGTVPVGRVETGVITPGMVVTFAPANVTTEVKSVEMHHQQLKEGVPGDNVGFNV
          	****.*********:***::.*********:*************:** :* *********

MG03641.1 	KNVSVKDIRRGNVAGDSKNDPPMGCASFNAQVIILNHPGQVGAGYAPVLDCHTAHIACKF
NCU02003.1	KNVSVKDIRRGNVAGDSKNDPPAGAASFTAQVIVLNHPGQVGAGYAPVLDCHTAHIACKF
FG08811.1 	KNVSVKDIRRGNVAGDSKNDPPMGAASFTAQVIVLNHPGQVGAGYAPVLDCHTAHIACKF
AN4218.1  	KNVSVKEIRRGNVAGDSKNDPPAGAASFTAQVIVLNHPGQVGAGYAPVLDCHTAHIACKF
          	******:*************** *.***.****:**************************

MG03641.1 	SEILEKLDRRTGKSIESNPKFIKSGDAAIVKMIPSKPMCVETFSEYPPLGRFAVRDMRQT
NCU02003.1	AELLEKIDRRTGKAVEASPKFIKSGDAAIVKMIPSKPMCVEAFTDYPPLGRFAVRDMRQT
FG08811.1 	AEIQEKIDRRTGKATEAAPKFIKSGDSAIVKMVPSKPMCVEAFTDYPPLGRFAVRDMRQT
AN4218.1  	AELQEKIDRRTGKSVESSPKFIKSGDAAIVKMIPSKPMCVESFTDYPPLGRFAVRDMRQT
          	:*: **:******: *: ********:*****:********:*::***************

MG03641.1 	VAVGVIKSVDKSQGTQGK0VTKSAAKAAKK
NCU02003.1	VAVGVIKAVDKSTAAAGK~VTKSAAKAAKK
FG08811.1 	VAVGVIKAVEKSTGAAGK~VTKSAAKAGKK
AN4218.1  	VAVGVVKSVEKSTAGAGK~VTKAAQKAGKK
          	*****:*:*:** .  ** ***:* **.**
```
